# Molecular Mechanism of Aggravation of Hypertensive Organ Damages by Short-Term Blood Pressure Variability

**DOI:** 10.2174/1573402111666141217112655

**Published:** 2015-09

**Authors:** Hisashi Kai, Hiroshi Kudo, Narimasa Takayama, Suguru Yasuoka, Yuji Aoki, Tsutomu Imaizumi

**Affiliations:** 1 Department of Internal Medicine, Division of Cardio-Vascular Medicine, Kurume University School of Medicine, Kurume, Japan;; 2 International University of Health and Welfare, Fukuoka Sannno Hospital, Fukuoka, Japan

**Keywords:** Angiotensin II, cardiac hypertrophy, fibrosis, inflammation, mineralocorticoid receptor, statins

## Abstract

There is increasing evidence that not only the elevation of systolic and diastolic blood pressure (BP) but also the increase in BP variability (or fluctuation) are associated with hypertensive organ damages and the morbidity and mortality of cerebrovascular and cardiovascular events. However, the molecular mechanism whereby the increase in BP variability aggravates hypertensive organ damages remains unknown. Thus, we created a rat chronic model of a combination of hypertension and large BP variability by performing bilateral sino-aortic denervation in spontaneously hypertensive rat. A series of our studies using this model revealed that large BP variability induces chronic myocardial inflammation by activating local angiotensin II and mineralocorticoid receptor systems and thereby aggravates cardiac hypertrophy and myocardial fibrosis, leading to systolic dysfunction, in hypertensive hearts. In addition, large BP variability induces the aggravation of arteriolosclerotic changes and ischemic cortical fibrosis in hypertensive kidney via local angiotensin II system.

## INTRODUCTION

For a long time, treatment of hypertension has been targeting the reduction in the “levels” of systolic and diastolic blood pressure (BP) to prevention of hypertensive organ damages, complications, and cerebrovascular and cardio-vascular events [1-3]. BP has several kinds of fluctuation in nature: for example, short-term (beat-to-beat and minute-to-minute) variability, circadian BP variation, visit-to-visit variability of office BP, and the seasonal BP variation [4-8]. Recent studies have demonstrated that various kinds of BP variability are independent risk of the progression of hyper-tensive organ damages and cerebrovascular and cardiovascular events as well as cognitive dysfunction [9-18]. However, it remains unknown whether the increase in BP variability is the cause or result of hypertensive organ damages and whether it should be taken into account as a new target in hypertension treatment. 

**Fig. (1):**
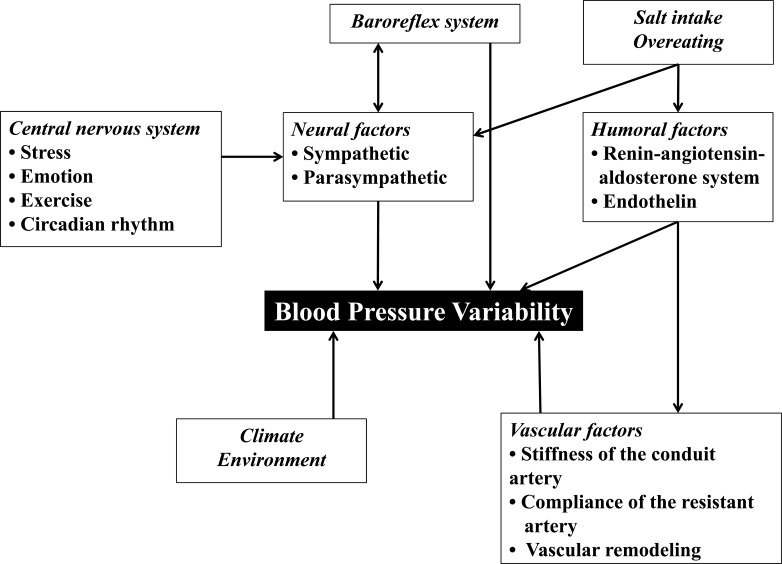
Regulating factors of blood pressure variability

Aggravation of BP variability is attributable to several factors [19] (Fig. **1**): Vascular factors include increase in the stiffness of the conduit arteries (i.e. the aorta and its major branches) by aging, hypertension, and atherosclerosis, decrease in the compliance of the resistant arteries/arterioles by hypertensive remodeling, and increase in vascular tonus of the resistance arteries/arterioles by increased sympatheticnerve activity and/or humoral factors, such as angiotensin II and endothelin. The vascular factors may be the largest cause of aggravation of BP variability in hypertension patients, especially in elderly patients, patients with long hypertension history, and patients with advanced atherosclerosis. Another important factor is neural factors, which mainly play a role in the buffering mechanism of the beat-to-beat BP variability mediated by the reflex loop consisting of the baroreceptors, the brain stem vasomotor center, and the afferent and efferent sympathetic/parasympathetic nerve systems. The neural factors are impaired by aging, diabetes, and neuropathies. In addition, many other factors, such as the central nervous system, mental stress, the climate/environmental factors, the humoral factors, etc., directly and/or indirectly affect BP variability exacerbation. Accordingly, it is not simple to investigate the mechanism of the large BP variability-induced aggravation of hypertensive organ damages, because the various co-existing, confounding factors should be eliminated. 

In this review article, we summarized a series of our studies demonstrating that chronic inflammation mediated by local angiotensin II system and mineralocorticoid receptor (MR) system plays a role in the mechanism whereby large BP variability aggravates hypertensive organ damages using a rat chronic model of a combination of hypertension and large BP variability.

## CHRONIC MODEL OF A COMBINATION OF HYPERTENSION AND LARGE BP VARIABILITY

**Fig. (2):**
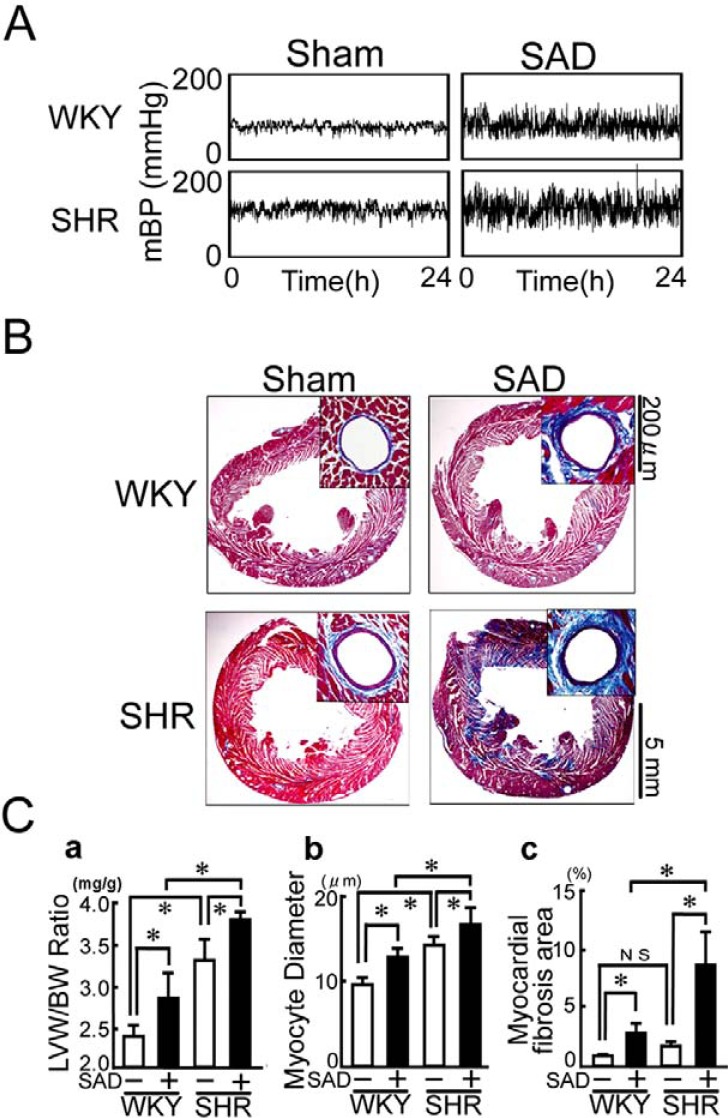
Chronic model of a combination of hypertension and large blood pressure variability. A. 24-h records of mean blood pressure (mBP) under the unrestricted, conscious condition. B. Representative microphotographs of Mallory-Azan-stained left ventricles. C. Pooled data of changes in myocyte diameter (a), %myocardial fibrosis area (b), and left ventricular (LV) fractional shortening (c). WKY, Wistar Kyoto rat; SHR, spontaneously hypertensive rat; SAD, bilateral sino-aortic denervation. Modified from Kudo *et al* [20].

We have created a chronic model of a combination of hypertension and large BP variability by performing bilateral sino-aortic denervation (SAD) in spontaneously hypertensive rat (SHR) [20]. Bilateral SAD consists of surgical resection of bilateral aortic depressor nerve, superior laryngeal nerve, superior cervical ganglia, and cervical sympathetic trunks and chemical ablation of the carotid and aortic arch baroreceptors [21,  22]. A transient activation of sympathetic nerve system and renin-angiotensin system was observed immediately after SAD. However, these changes were normalized within a couple of weeks and circulating levels of catecholamines, active renin, angiotensin II, and aldosterone were similar to those of sham-operated SHR 7 weeks after the operation. In the chronic stage of SAD (7 weeks after SAD), the standard deviation of mean BP, an indicator of BP variability, was increased by approximately two-fold in SHR+SAD, as compared with SHR+sham, whereas the average of mean BP did not differ between the two groups (Fig. **2A**) [20]. Therefore, SHR+SAD is regarded as a chronic model of a combination of hypertension and large BP variability without activation of systemic sympathetic nerve system and renin-angiotensin-aldosterone system [23]. 

## EFFECTS OF LARGE BP VARIABILITY ON HYPER-TENSIVE CARDIAC REMODELING

SHR shows hypertensive cardiac remodeling characterized by myocyte hypertrophy and perivascular fibrosis. SAD aggravated hypertensive cardiac remodeling (Fig. **2B** and **2C**) [20]. The effect of mean BP elevation and BP variability enlargement was apparently additive on cardiomyocyte hypertrophy (Fig. **2B** and **2Ca**). In contrast, the effect on myocardial fibrosis was synergetic: Large BP variability not only enhanced perivascular fibrosis but also induced patchy and massive interstitial fibrosis (Fig. **2B** and **2Cb**). Echocardiographic examination revealed left ventricular systolic dysfunction in SHR+SAD (Fig. **2C**). These findings suggest that large BP variability aggravates hypertensive myocardial damages and thereby induced myocyte death, resulting in replacement/reparative myocardial fibrosis and consequently systolic dysfunction.

## CHRONIC INFLAMMATORY CHANGES INDUCED BY CARDIAC ANGIOTENSIN SYSTEM

**Fig. (3):**
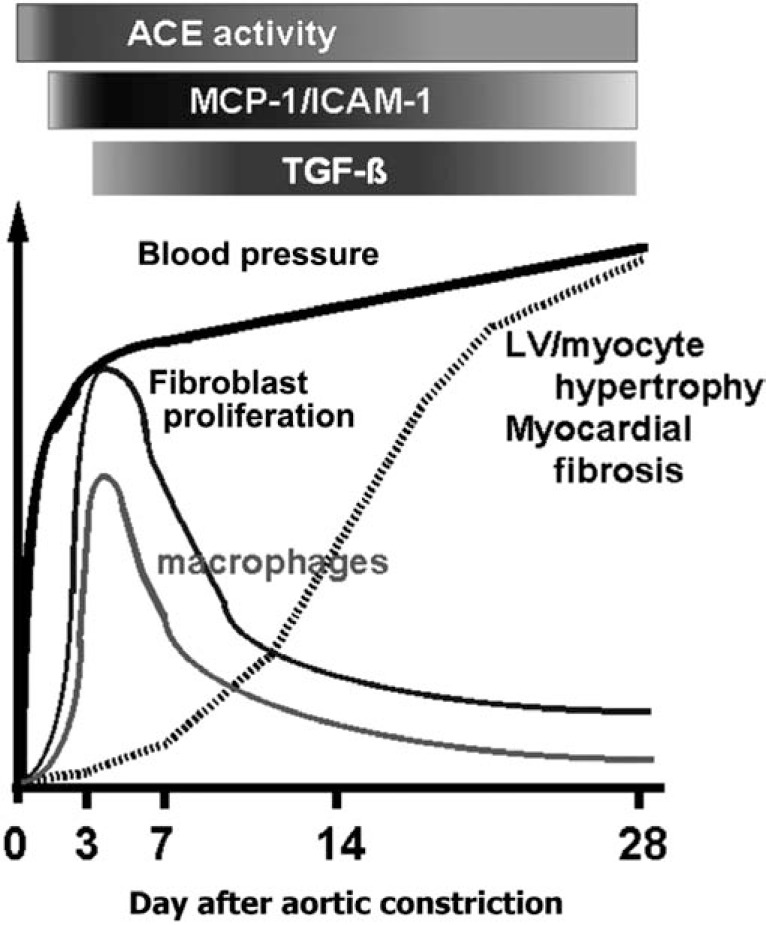
Scheme of the inflammatory changes in a rat model of hypertensive heart with diastolic, but not systolic, dysfunction induced by abdominal aortic constriction. ACE, angiotensin-converting enzyme; MCP-1, macrophage chemoattractant factor-1; TGF-β, transforming growth factor-β. Modified from Kai *et al* [29].

Previously, we have demonstrated that a rapid BP elevation induced by abdominal aortic constriction transiently induces activation of myocardial angiotensin-converting enzyme activity, induction of monocyte chemoattracting protein-1 (MCP-1) and transforming growth factor-β (TGF-β), and macrophage infiltration in the myocardium, especially in the perivascular area around the intramyocardial coronary arterioles, in normotensive WKY rats (Fig. **3**) [24,  32]. Moreover, the angiotensin II-mediated inflammation triggered myocyte hypertrophy and perivascular fibrosis, resulting in diastolic dysfunction with preserved systolic function [24,  28]. Thus, we hypothesized that repeated rapid BP rise may induce repetitive, chronic inflammation in hypertensive heart when BP variability is exacerbated.

**Fig. (4):**
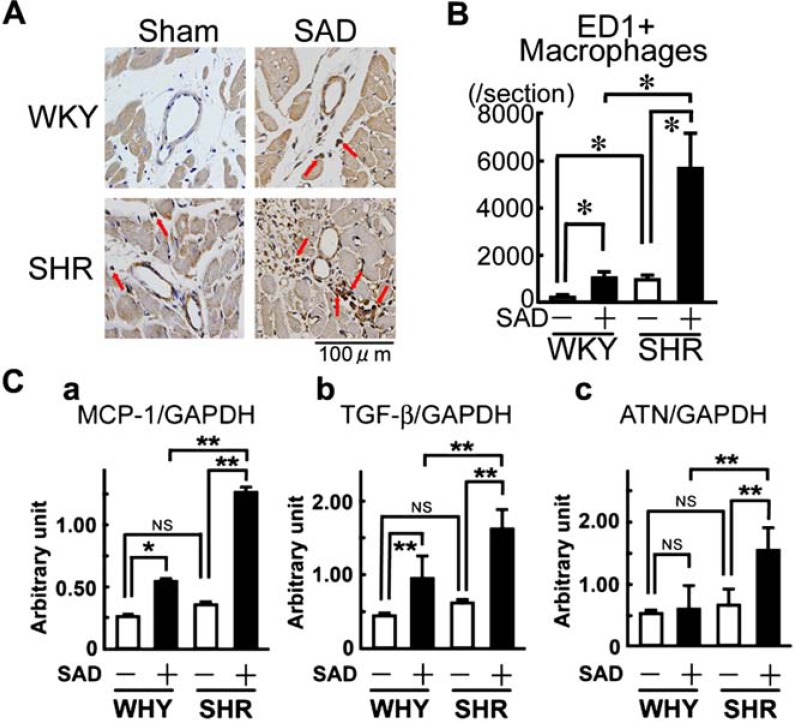
Scheme of the inflammatory changes in a rat model of hypertensive heart with diastolic, but not systolic, dysfunction induced by abdominal aortic constriction. ACE, angiotensin-converting enzyme; MCP-1, macrophage chemoattractant factor-1; TGF-β, transforming growth factor-β. Modified from Kai *et al* [29].

BP elevation (SHR+sham) alone and large BP variability alone (WKY+SAD) had mild MCP-1 and TGF-β upregulation and macrophage infiltration in hypertensive heart (Fig. **4**). In contrast, SHR+SAD showed extensive increase in MCP-1 and TGF-β expression and massive macrophage infiltration [20]. Moreover, myocardial angiotensinogen was markedly upregulated in SHR+SAD. Thus, it is suggested that chronic inflammation mediated by local angiotensin II system contributes to the aggravation of hypertensive cardiac remodeling induced by large BP variability.

**Fig. (5):**
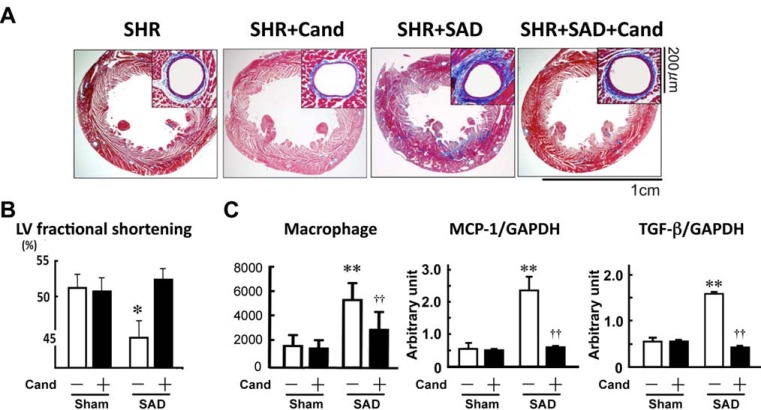
Effects of non-depressor dose of candesartan (Cand) on the large blood pressure variability-induced hypertensive cardiac remodeling (A), left ventricular (LV) fractional shortening (B), and inflammatory changes (C). SHR, spontaneously hypertensive rat; SAD, bilateral sinoaortic denervation; MCP-1, macrophage chemoattractant factor-1, TGF-β, transforming growth factor-β. Modified from Kudo et al [20]

Non-depressor dose of an angiotensin II type I receptor blocker (ARB), candesartan, did not change BP variability as well as the mean BP, in SHR+SAD. However, the small dose of candesartan inhibited the large BP variability-induced cardiac remodeling, i.e. cardiac hypertrophy and myocardial fibrosis, and resultantly prevented systolic dysfunction in SHR (Fig. **5A** and **5B**). Cardiac MCP-1 and TGF-β induction and macrophage infiltration induced by large BP variability were also prevented by non-depressor dose of candesartan (Fig. **5C**). These findings indicate that local angiotensin II system activation is attributable to the mechanism of aggravation of hypertensive cardiac remodeling induced by large BP variability.

## CHRONIC INFLAMMATORY CHANGES INDUCED BY CARDIAC MINERALOCORTICOID RECEPTOR

**Fig. (6):**
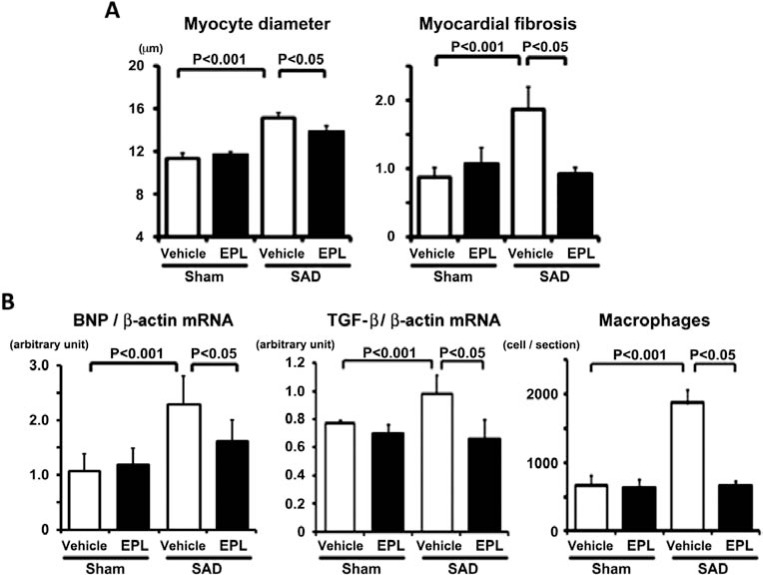
Effects of non-depressor dose of eplerenone (EPL) on the large blood pressure variability-induced hypertensive cardiac remodeling in SHR. A. Pooled data showing the changes in myocyte diameter and %myocardial fibrosis. B. Pooled data showing the changes in braintype natriuretic peptide (BNP) mRNA expression (a) and transforming growth factor-β (TGF-β) mRNA expression (b), and macrophage infiltration (c). SAD, bilateral sino-aortic denervation. Modified from Yasuoka et al [33].

In this chronic model of a combination of hypertension and large BP variability, activation of MR was documented in the intramyocardial coronary arterioles and, to a lesser extent, in the cardiac myocytes [33]. Non-depressor dose of eplerenone, a specific MR blocker, inhibited the large BP variability-induced MR activation, inflammatory changes, cardiac hypertrophy, and myocardial fibrosis and thereby prevented systolic dysfunction in SHR+SAD without changing BP variability (Fig. **6**). Because the circulating levels of aldosterone and corticosteroid were not changed, it is suggested that MR activation induced by large BP variability is the agonist-independent phenomenon. Accordingly, it is suggested that cardiac MR activation plays a role in the initiation of chronic inflammation and cardiac remodeling induced by large BP variability.

## CARDIOMYOCYTE HYPERTROPHY AND RHOA AND RAS/ERK SYSTEMS

**Fig. (7):**
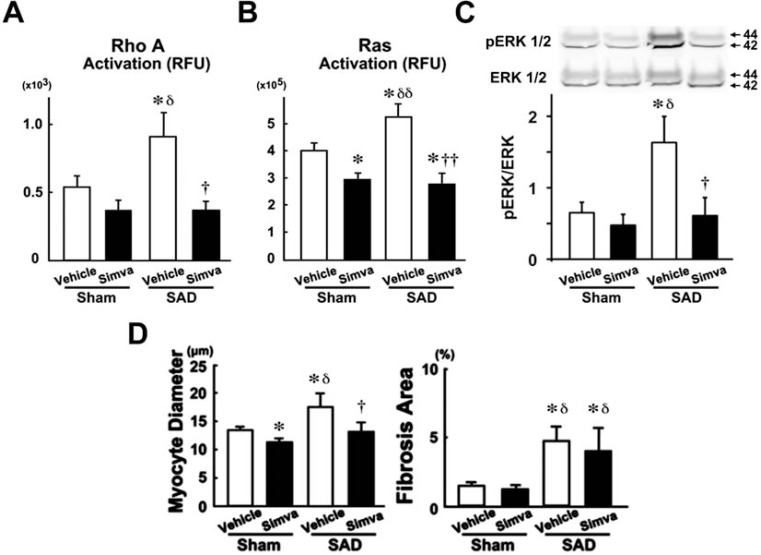
Effects of simvastatin (Simva) on the large blood pressure variability-induced RhoA activation (A), Ras activation (B), ERK1/2 phosphorylation (C), and cardiac remodeling (D). SHR, spontaneously hypertensive rat; SAD, bilateral sino-aortic denervation. Modified from Takayama *et al* [34].

It was suggested that activation of cardiac RhoA system and Ras/ERK system would be involved in cardiomyocyte hypertrophy induced by large BP variability [34]. Myocardial activity of RhoA and Ras and phosphorylation of ERK1/2 increased in SHR+SAD, as compared with SHR+sham (Fig. **7**). Simvastatin inhibited the activation of RhoA and RAS/ERK systems and then inhibited cardiac hypertrophy, but not myocardial fibrosis, in SHR+SAD without affecting BP variability. Thus, it is suggested that RhoA and Ras/ERK systems contribute to the mechanism of cardiac hypertrophy induced by large BP variability in hypertensive heart.

## RENAL DAMAGES INDUCED BY LARGE BP VARIABILITY

**Fig. (8):**
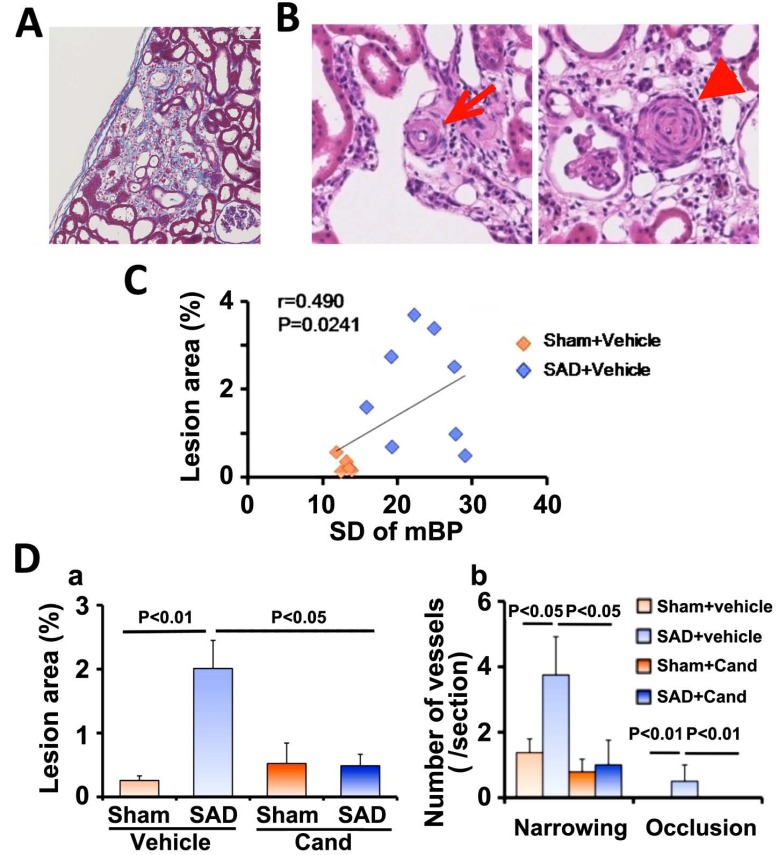
Renal cortex damage induced by a combination of hypertension and large blood pressure variability. A. Representative microphotograph of hematoxylin-eosin stained renal cortex section showing focal ischemic sclerotic lesion. B. Representative microphotographs of hematoxylin-eosin stained renal cortex sections showing vascular wall thickening with luminal narrowing (arrow, left panel) and occlusion (arrow head, right panel) of the pre-glomerular arterioles. C. Correlation between the extent of BP variability, standard deviation of mean blood pressure (SD of mBP), and %ischemic sclerotic lesion area. D. Pooled data showing the effects of non-depressor dose of candesartan (Cand) on the ischemic sclerotic lesion area (a) and the number of the arteriolosclerotic lesions (b). SAD, bilateral sino-aortic denervation. Modified from Aoki *et al* [35].

BP variability aggravates hypertensive renal damages as well. Large BP variability induced patchy, wedge-shaped, focal sclerotic lesions accompanied by interstitial fibrosis and ischemic changes of the glomeruli and tubules in the renal cortex in SHR (Fig. **8A**) [35]. The pre-glomerular arterioles adjacent to the cortical sclerotic lesions had arteriolosclerotic changes characterized by vascular smooth muscle cell proliferation and extracellular matrix deposition, leading to the luminal narrowing and occlusion (Fig. **8B**). The area of the fibro-ischemic lesions was significantly associated with the extent of BP variability (Fig. **8C**). Non-depressor dose of candesartan prevented the cortical sclerotic lesions and arteriolosclerotic changes in SHRs with SAD without changing BP variability (Fig. **8D**). Because systemic renin-angiotensin-aldosterone system is not activated, [23] these findings suggest that local angiotensin system mediates large BP variability-induced pre-glomerular arteriolosclerosis and the resultant cortical ischemic sclerotic changes in hypertensive kidney, which would lead to the progression of chronic kidney disease.

## STRAIN VESSEL HYPOTHESIS AND BP VARIABILITY

Recently, Ito *et al*. have proposed “strain vessel” hypothesis for the mechanism of hypertensive organ damages at the early stage [36]. Briefly, generic vessels, most of the systemic arterioles, become smaller gradually with branch off, and the hemodynamic strain onto the vascular wall also declines gradually, as BP decreases. It is the case with the intralobular and afferent arterioles to the majority of nephrons, i.e. the superficial cortical nephrons. In contrast, the strain vessels, such as the penetrating arteries in the brain and the interlobular and afferent arterioles to the juxtamedullary nephrons, branch off directly from large vessels. Given direct exposure to high BP, the strain vessels are the primary sites of accelerated progression of hypertensive damages. Thus, the strain vessel hypothesis is an attractive explanation why the penetrating artery is the common site of lacunar infarction and hypertensive cerebral bleeding and why the juxtamedullary nephrons are the primary region of albuminuria.

It is interesting that the pre-glomerular arteriosclerotic lesions were mostly found in the juxtamedullary cortex in SHR+SAD. This finding raises the possibility that the strain vessels would be the initial target of the large BP variability-induced renal damages as well. It is plausible that the strain vessels are directly exposed to the impact of the repetitive BP fluctuation in addition to high mean BP, which would aggravate further hypertensive organ damages. Here, it was reported that large day-by-day or visit-to-visit variability was an independent risk for strokes [13,  14]. Therefore, it is possible that large BP variability-induced aggravation of hypertensive damages of another “strain vessel”, 

the penetrating artery [36], which is the common site of hypertensive cerebral bleeding and lacunar infarction, would be in part the cause of the increased risk of strokes.

## CONCLUSIONS AND CLINICAL IMPLICATIONS

**Fig. (9):**
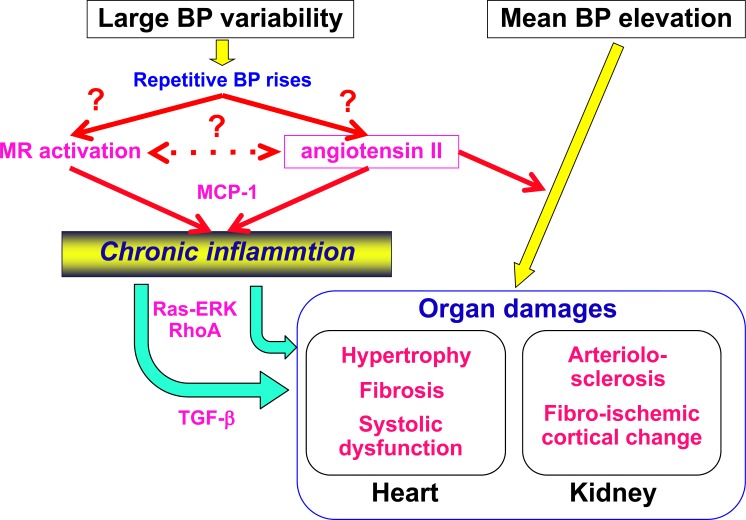
Scheme of proposed molecular mechanism of the large blood pressure (BP) variability-induced aggravation of hypertensive organ damages in the heart and kidney. MR, mineralocorticoid receptor; MCP-1, macrophage chemoattractant factor-1, TGF-β, transforming growth factor-β.

Activation of local angiotensin II system and MR system is the molecular mechanism whereby large BP variability aggravates hypertensive organ damages in the heart and kidney (Fig. **9**). Currently, the initial mechanism whereby large BP variability activates local angiotensin II system and MR system remains undetermined. And, “strain vessel” may be the main target of large BP variability, suggesting the possible rationale for intervention to BP variability for prevention of stroke and chronic kidney disease as well as heart failure. These issues should be addressed in future study. 

Current guidelines for hypertension treatment recommend the combination therapy of an ARB and a calcium channel blocker and/or diuretics in order to lower BP levels effectively and to reduce cerebrovascular and cardiovascular events [1-3]. Several meta-analyses and systematic reviews of antihypertensive agents have demonstrated that calcium channel blockers and diuretics have the ability to reduce visit-to-visit BP variability but ARB does not [37-39]. However, our studies have shown that ARB prevents molecular mechanism of the aggravation of hypertensive organ damages induced by large BP variability, independent of BP level and BP variability. Taken together, it is expected that the combination of ARB, which has “the effects beyond BP variability lowering”, and calcium channel blocker and/or diuretics, which have “BP variability lowering effects”, would have further beneficial effects on prevention of hyper-tensive organ damages and resultantly, cerebrovascular and cardiovascular events.

## CONFLICT OF INTEREST

H Kai has received lecturer fees from TAKEDA (Osaka, Japan).
